# Acute atorvastatin treatment restores the cardioprotective effects of ischemic postconditioning in hyperlipidemic rats

**DOI:** 10.18632/oncotarget.19232

**Published:** 2017-07-14

**Authors:** Tao Sun, Hong-Ju Zhang, Chayakrit Krittanawong, Su Wang, Ying Tao, Zhao Li, Qiancheng Yin, Donghua Zhang, Qian Wang, Ji Huang, Jingmei Zhang, Zhizhong Li, Yutong Cheng

**Affiliations:** ^1^ Department of Cardiology, Beijing Anzhen Hospital, Capital Medical University, Beijing, China; ^2^ Division of Ultrasound, Fu Wai Hospital, National Center for Cardiovascular Diseases, Beijing, China; ^3^ Department of Internal Medicine, Icahn School of Medicine at Mount Sinai St. Luke's and Mount Sinai West, New York, NY, USA

**Keywords:** ischemic postconditioning, statin, atorvastatin, hyperlipidemia, infarct size-limiting effect

## Abstract

**Background:**

Ischemic Postconditioning (IPC) reduces ischemia/reperfusion (I/R) injury under normal conditions. HMG-CoA reductase inhibitors (statins), which inhibit the synthesis of mevalonate, can interfere with the cardioprotective effect of IPC. However, the beneficial role of IPC in hyperlipidemic patients, post-acute administration of statins remains unknown. This study was to determine if acute administration of atorvastatin affect the infarct size-limiting effect of IPC in hyperlipidemic rats.

**Results:**

Compared to control group, infarct size decreased more significantly in atorvastatin+IPC and atorvastatin+IPC+wortmannin groups than IPC or atorvastatin+IPC+PD98059 groups. Phosphorylation of PI3K/Akt was attenuated in atorvastatin + IPC+ wortmannin group, phosphorylation of P42 MAPK/ERK was increased in atorvastatin+IPC and atorvastatin+IPC+wortmannin groups.

**Materials and Methods:**

Ninety four-weeks old male SD rats fed with cholesterol enriched diet for six weeks were randomized into nine groups (*n* = 10/group) - sham group, control group, IPC group, atorvastatin group, wortmannin group, PD98059 group, atorvastatin+IPC group, atorvastatin+IPC+wortmannin group and atorvastatin+IPC+PD98059 group. Atorvastatin was administered orally 12 hours before myocardial reperfusion.

**Conclusions:**

Post-translational activation of P42 MAPK/ERK, rather than PI3K/Akt, participates in the net protective effect of IPC and atorvastatin in hyperlipidemia.

## INTRODUCTION

Ischemic heart disease is the leading cause of death in the industrialized world. Myocardium reperfusion can lead to myocardial infarction, cardiac arrhythmias, and contractile dysfunction [1]. Numerous studies have confirmed the myocardial protective effect of IPC since its first demonstration more than a decade ago [2]. The majority of these studies were performed in animal models, in which ischemia/reperfusion was imposed in the absence of comorbidities [1]. However, myocardium ischemic victims often have simultaneous classic cardiovascular risks, inclusive of hypertension, hyperlipidemia, diabetes and heart failure, which markedly interfere with the cardioprotective effect of IPC. It has been shown that the infarct size-limiting effect of IPC is attenuated in hyperlipidemic rabbits [3, 4] and cholesterol-fed rats [5].

The 3-hydroxy-3-methylglutaryl coenzyme, a reductase inhibitor widely known as statins, inhibits the synthesis of mevalonate. Statin has been strongly recommended in hyperlipidemic patients and has been shown to reduce the risk of cardiovascular mortality [6]. Numerous studies have evaluated the non-canonical (cholesterol-independent) positives of acute statin administration. Simvastatin administration for three weeks limited the infarct size both in normal and in hyperlipidemic rabbits, irrespective of IPC [7]. Furthermore, combination of acute atorvastatin with IPC had a stronger protective effect within the hearts of diabetic rats; in comparison, chronic statin with IPC failed to protect hearts against reperfusion injury in either diabetic or non-diabetic rats [8]. When IPC was combined with acute atorvastatin treatment, it inhibited the cardioprotective effects of IPC. Treatment with chronic atorvastatin in isolated rat heart was shown to downregulate IPC-mediated post translational activation of endothelial nitric oxide synthase (eNOS) and Akt [9]. The ARMYDA-ACS trial [10] confirmed this findings further, in which hypercholesterolemia was a common risk factor.

Therefore, defining the underlying molecular mechanisms of statin administration on the cardioprotective effect of IPC in hyperlipidemia is of significant clinical significance. Hence, the objective of the current study was to determine if acute administration of atorvastatin potentiates the cardioprotective effect of IPC in hyperlipidemic rats and whether such potentiation occurs through the pro-survival PI3K/Akt and/or P42 MAPK/ERK signaling pathways.

## RESULTS

### Infarct size

**Figure 1:**
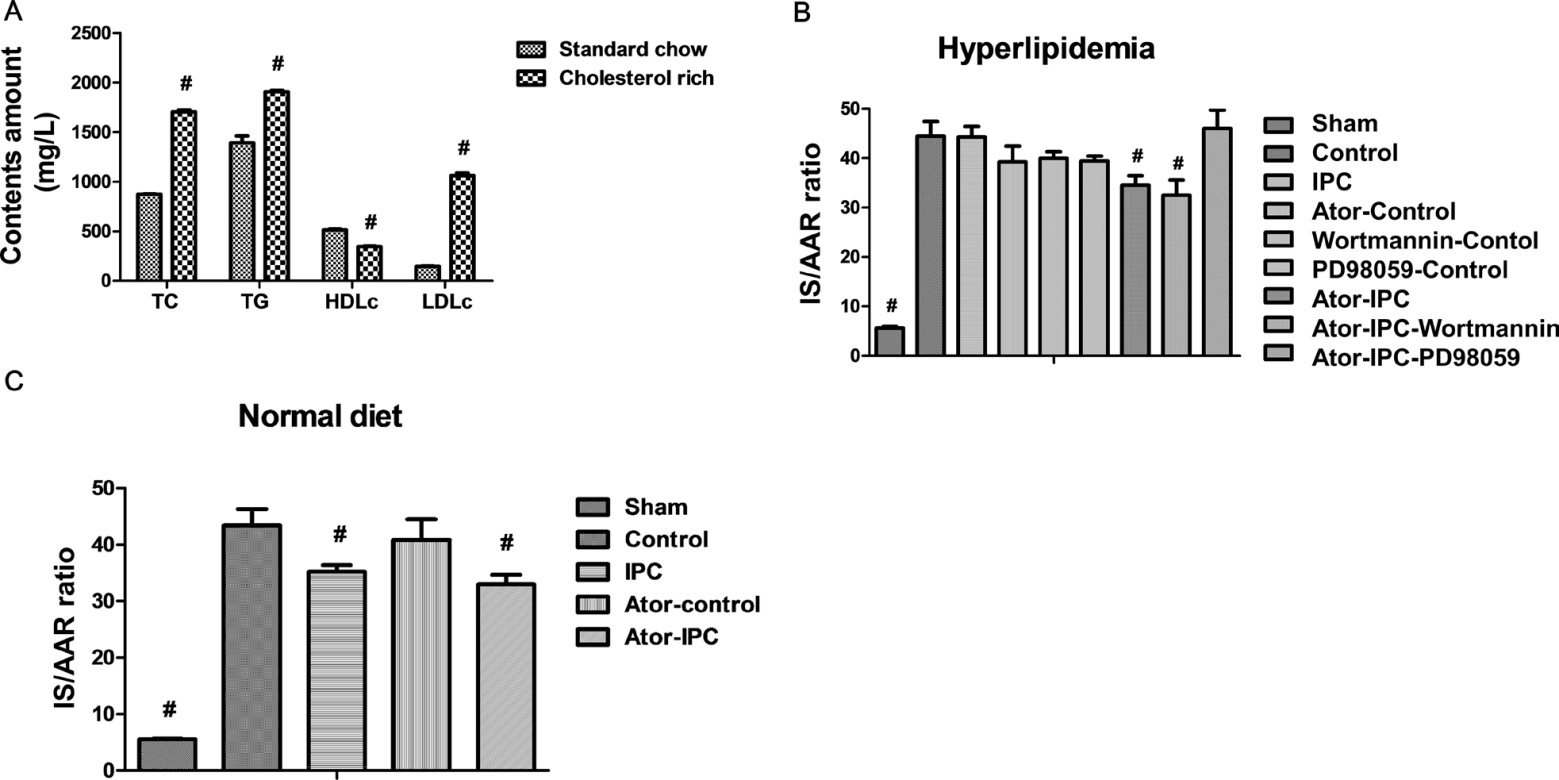
Atorvastatin treatments protect myocardial functions after IPC (**A**) The hyperlipidemic rats displayed increased TC, TG and LDLc levels but decreased HDLc levels compared with normal rats. (**B**) Myocardial infarction size (IS) was determined by triphenyltetrazolium chloride (TTC) staining after the reperfusion and expressed as percentage of area at risk (AAR). (**C**) The IS/AAR is decreased in normal diet rats with Atorvastatin treatments after IPC. ^#^*P* < 0.05 versus control group.

To establish a hyperlipidemic animal model, rats were fed with normal food or cholesterol rich diet. The hyperlipidemic rats displayed increased TC, TG and LDLc levels but decreased HDLc levels compared with normal rats (sham group) (Figure 1A). In the hypercholesterolemia rats, I/R ratio between myocardial infarction sizes (IS) and area at risk (AAR) was not significantly different between the control (I/R group in which the rats were fed with cholesterol enriched diet) and IPC groups. However, Atorvastation+IPC and Atorvastatin+IPC+wortmannin groups had significantly lower I/R versus the control group (Figure 1B; *P* < 0.001 in either case). The difference was not significant between Atorvastatin+IPC+PD98059 and control groups (Figure 1B). To exclude the possibility that Atorvastatin alone could protect cardiovascular functions, we also treated the hyperlipidemic rats with Atorvastatin alone, wortmannin alone, or PD98059 alone in a non-IPC control condition. Our results show the atorvastatin decreases I/R ratio only slightly, but not at statistical significant levels, indicating atorvastatin specifically restores the cardioprotective effects during ischemic postconditioning. The wortmannin alone, or PD98059 alone treatments did not decrease the I/R ratio (Figure 1B). In addition, our results show the atorvastatin treatments could protect against infarction in normal diet rats as well (Figure 1C).

### Reduction of infarct sizes by statin were independent of plasma cholesterol levels

Plasma cholesterol levels were determined following manufacturer guidelines (Diasys Diagnostic Systems GmbH, Holz-Heim, Germany). Plasma LDL levels were determined spectrophotometrically using the Cholesterol LDL direct kit (Biosystems S.A., Spain). Cholesterol levels did not exhibit significant differences among the different experimental groups (sham group, 786 ± 121; control group, 780 ± 125; IPC, 860 ± 165; Atorvastatin+IPC, 810 ± 145; Atorvastatin+IPC+wortmannin, 790 ± 138; Atorvastatin+IPC+PD98059, 820 ± 122 mg.dL^−1^), indicating that reduction of infarction sizes observed in the Atorvastatin+IPC and Atorvastatin+IPC+wortmannin groups were independent of any potential plasma cholesterol-lowering effects of atorvastatin.

### Effect of statin is mediated by phospho P42 MAPK/ERK

**Figure 2:**
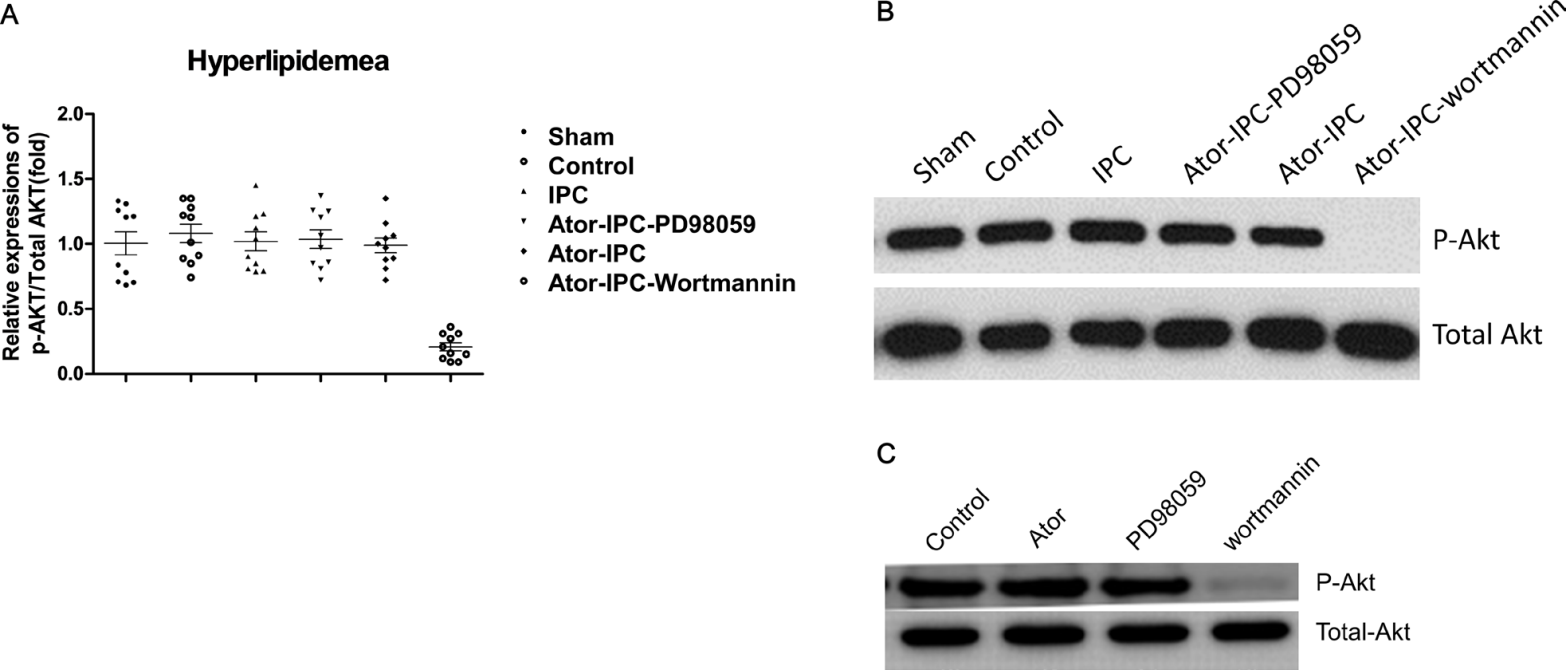
Phosphorylation of Akt is unchanged with atorvastatin treatments after IPC (**A**) The phosphorylation of Akt was detected in ten rats of each group. The results were analyzed as the ratio of p-Akt to total Akt. (**B**) Representative immunoblots demonstrating phosphorylated and total protein levels of Akt in the indicated treatment groups. (**C**) Representative results showing the phosphorylation of Akt detected by immunoblots in atorvastatin alone, PD98059 alone and Wortmannin alone group.

Steady state levels of total Akt and P42 MAPK/ERK as determined by immunoblot analysis was unaffected in the different experimental groups. The phosphorylation of Akt in the Atorvastatin+IPC+wortmannin group decreased significantly (Figure 2A, 2B). Atorvastatin alone or PD98059 alone did not change the phosphorylation of Akt and total Akt; only wortmannin treatments suppressed the phosphorylation of Akt (Figure 2C).

**Figure 3:**
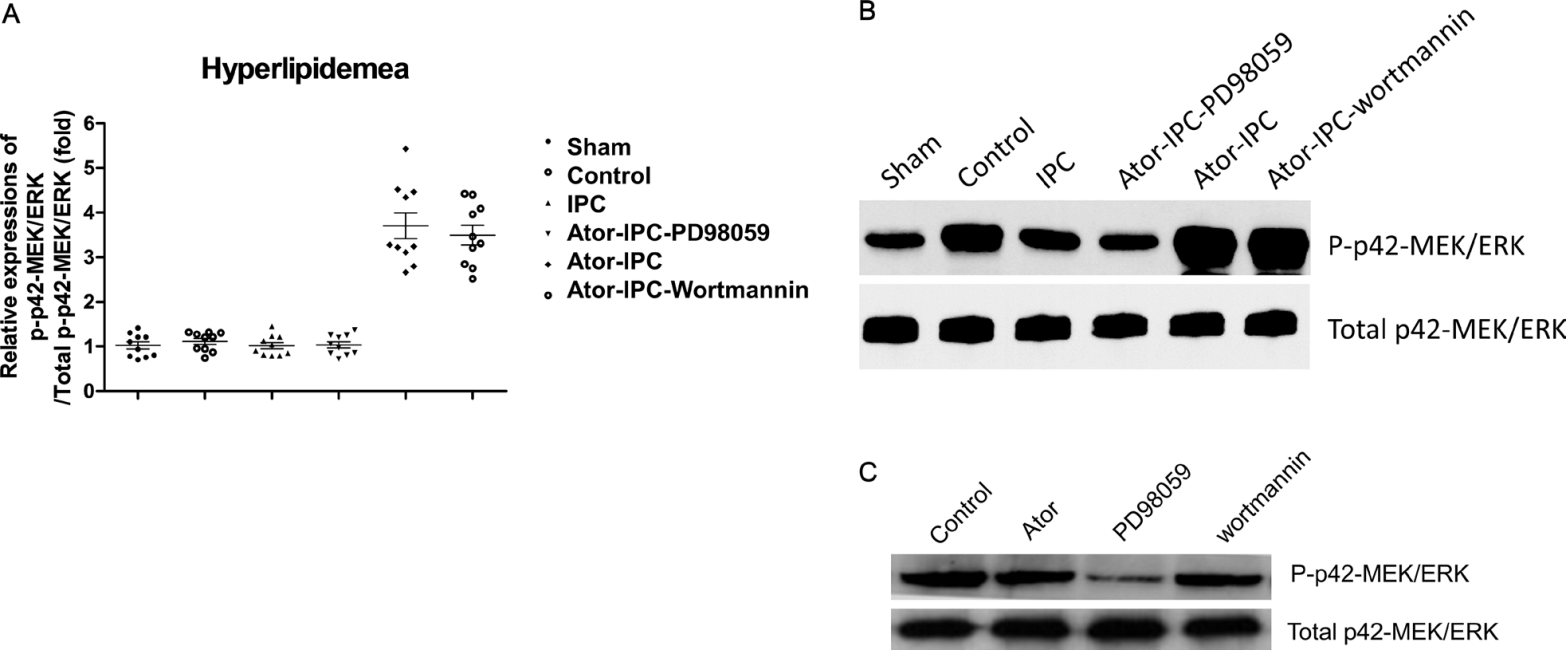
Phosphorylation of P42 MAPK/ERK is upregulated with the atorvastatin treatments after IPC (**A**) The phosphorylation of P42 MAPK/ERK was detected in ten rats of each group. The results were analyzed as the ratio of p-P42 MAPK/ERK to total P42 MAPK/ERK. (**B**) Representative immunoblots demonstrating phosphorylated and total protein levels of P42 MAPK/ERK in the indicated treatment groups. (**C**) Representative results showed the phosphorylation of P42 MAPK/ERK detected by immunoblots in atorvastatin alone, PD98059 alone and Wortmannin alone group.

However, the phosphorylation of P42 MAPK/ERK were increased significantly both in Atorvastatin+IPC and Atorvastatin+IPC+wortmannin groups, but decreased (though not statistically significant) in the Atorvastatin+IPC+PD98059 group (Figure 3A, 3B). Wortmannin or atorvastatin alone did not affect the phosphorylation of p42-MEK/ERK; which was inhibited in the PD98059 alone group (Figure 3C).

## DISCUSSION

Myocardium reperfusion may lead to myocardial infarction, cardiac arrhythmias, and contractile dysfunction [1]. IPC can reduce infarction size and provide cardioprotection, both in animals and human beings. Recent studies [5, 11] revealed that hyperlipidemia can increase the myocardial susceptibility to I/R injury. The 3-hydroxy-3-methylglutaryl coenzyme was shown to reduce cardiovascular mortality in hyperlipidemic patients [6]. Even though many studies evaluated at the cholesterol-independent protective effects of the statins [7–10], the exact mechanism of potentiating IPC is still largely inconclusive. Part of the discrepancy stems from the study model used, drug administration routes, and protocol adopted for IPC induction in these studies. However, this underlies the tremendous clinical significance of elucidating the molecular mechanism of cardioprotective synergism achieved by co-administration of IPC and statin in hyperlipidemic patients. Nevertheless, the effects provided in hyperlipidemic disorders by IPC when acute statin is administered and the potential mechanism via the activation of prosurvival kinases Akt and p42 MAPK/ERK are still inconclusive.

It was shown that cholesterol diet-induced hyperlipidemia impairs the cardioprotective effect of IPC, at least in part via deterioration of the IPC-induced early increase in peroxynitrite formation [5]. It was further shown that IPC reduced the infarct size in hyperlipidemic rats in comparison with I/R group and that this might be mediated by upregulation of HIF-1α [12]. In the current study, IPC alone failed to markedly reduce the infarction size compared with control group in hyperlipidemic rat model.

It was shown that acute administration of lovastatin attenuated Akt phosphorylation-mediated activation and increased the P42 MAPK/ERK phosphorylation in normal rats [13]. Involvement of ERK1/2, rather than PI3K/Akt, was shown to be more important in the reduction of infarct size achieved with IPC in normal rabbit hearts [14]. However, the inhibition of glycogen synthase kinase-3 in homozygous GSK-3 double knockout mice did not attenuate cardioprotective effect of IPC [15], indicating that the cardioprotective action of IPC is not mediated by PI3K/Akt signaling pathway. In contrast, it was shown that acute atorvastatin administration with IPC could recover contractile dysfunction in hearts from diabetic rats and that activated Akt signaling pathways could enhance these protective effects [8]. In summary, there is contrasting data that suggest that the cardioprotective effect of IPC is mediated by PI3K/Akt and P42 MAPK/ERK signaling pathways alone or in combination. In the current study, oral administration of atorvastatin leads to the limiting-size infarction in hyperlipidemic animals. Furthermore, the Atorvastatin+IPC group showed robust P42 MAPK/ ERK, but not Akt, phosphorylation, leading to the hypothesis that cardioprotection is based briefly on the activation P42 MAPK/ ERK instead of Akt pathway.

The dose of wortmannin [16] used in the current study was previously shown to be adequate in a similar model to suppress insulin-induced PI3K activation. Wortmannin, impaired cardioprotective effects in the signal transduction cascade, when co-treated with wortmannin, the myocardium protection in Atorvastatin+IPC+wortmannin group should theoretically be discounted. Even though, the area of risks showed no significant differences between control group and Atorvastatin+IPC+wortmannin group. The phosphorylated Akt on Ser473 significantly decreased in the Atorvastatin+IPC+wortmannin group compared to the control group. It was speculated that acute statin might activate the P42 MAPK/ ERK signal pathways in parallel, which will overcome the inhibitory effects of wortmannin. Compared to the control group, when PD98059 was applied, there was no limiting-size observed in the Atorvastatin+IPC+PD98059 group and the activation P42 MAPK/ ERK was decreased, but not significantly attenuated. In this study, we focused on the PI3K/Akt and P42 MAPK/ERK activation to explore the mechanisms of the role of IPC in hyperlipidemic rats and observed consistent high-and low variation between the area at risk and phosphorylation in P42 MAPK/ ERK instead of Akt. Consequently, we can conclude that P42 MAPK/ ERK activation rather than PI3K/Akt might participate in the overall protective effect of IPC in hyperlipidemic disorders when acute statin is administered.

ERK1/2 is a kinase in the mitogen-activated protein kinase (MAPK) family and phosphorylation of ERK (p-ERK) can be used as a common end point measurement for the activation of many classes of G protein coupled receptors (GPCR). PI3 kinase, Akt and ERK were termed “reperfusion injury survival kinases” (RISK). The tight coupling between these kinases and mPTP formation was demonstrated in rat cardiomyocyte model [17]. It is believed that RISK act to prevent mPTP formation in the reperfused heart. Previous study has suggested that the activation of ERKs protected cardiomyocytes during ischemia/reperfusion [18]. Immunoblot analysis with a phosphospecific ERK (Thr 202/Tyr 204) antibody showed a decreased content of cytosolic phospho-ERK-1 and phospho-ERK-2 [18], similar to what we observed in our study. So the increased content of phosphorylated ERK may prevent mPTP formation to protect heart from ischemic and reperfusion injury in post conditioning model. However, one important limitation of our study is we did not probe for the proteins downstream of ERK, which will be needed in future studies.

To summarize, this study demonstrated that acute statin can restore the cardioprotective effect of IPC in hyperlipidemic rats through activation of ERK1/2 rather than PI3K/Akt signaling pathway.

## MATERIALS AND METHODS

### Animal

All animal studies conformed to the National Institutes of Health Guide for the Care and Use of Laboratory Animals (NIH Pub. No. 85–23, Revised 1996) and was approved by the An Zhen Hospital ethics committee.

### Surgical intervention protocol

Myocardial ischemia-reperfusion was induced following a coronary occlusion protocol as described before [19].

### Experimental Grouping and Treatment

Four-weeks old male Sprague-Dawley (SD) rats with an average weight of 200˜250g were provided by Animal Center of Anzhen Hospital, Capital Medical University. Eighty SD rats fed cholesterol enriched diet (Table 1) for six weeks were randomized into eight experimental groups (*n* = 10 in each group) - control group, IPC group, atorvastatin alone group, wortmannin alone group, PD98059 group, atorvastatin+IPC group, atorvastatin+IPC+wortmannin group, and atorvastatin+IPC+PD98059 group. In order to prove that the high-fat diet can induce hyperlipidemia, we also had a normal diet (sham) group (*n* = 10). IPC was induced by five cycles of 30-seconds ischemia/reperfusion following 30 minutes myocardial ischemia. The rats were subjected to 30-minutes myocardial ischemia followed by five cycles of 30-seconds ischemia/reperfusion. Atorvastatin sodium (3.0 mg/kg of body weight) [20] (kindly donated by Elpen Pharmaceutical Co. Inc.) was administered by a feeding tube once 12 hours before the surgical intervention.

**Table 1:** Ingredients of the standard rodent chow and the cholesterol enriched diet used in the current study

Ingredients / Kind of diet	Standard rodent chow (%)	The cholesterol enriched diet (%)
Normal feedstuff	100	90
Cholesterol	0	2
Lard	0	7.5
Pig choline	0	0.5

The energy values of triglyceride and the cholesterol were 38 kJ/g and 30 kJ/g, respectively.

The effects of atorvastatin were investigated after inhibition of PI3K with wortmannin (15 μg kg^−1^, i. v., *n* = 5) for 15 minutes prior to reperfusion. The Atorvastatin+IPC+PD98059 group was treated with the PD98059 (15 mg kg^−1^, i. v., *n* = 6), a non-ATP competitive MEK inhibitor, for 15 minutes prior to reperfusion. The doses of PD98059 and wortmannin have been shown to be effective in similar models [21]. For the individual control groups drug or inhibitor administration were done at doses same as when used in combination with another regimen as mentioned above.

At the end of the treatment, plasma was obtained from 3 ml of blood through centrifugation. Plasma samples were stored at −80°C for subsequent total cholesterol measurements. Cardiac tissues were either frozen in liquid nitrogen or alternatively prepared for measurement of infarct size.

### Measurements of total cholesterol, triglycerides, LDL and HDL

The measurements of TC (STA-384), TG (STA-396), LDL and HDL (STA-391) were performed using assay kits from Cell Biolabs (San Diego, CA USA) according to the manufactory's instruction.

### Risk area and infarct size measurement

After the end of reperfusion, hearts were harvested and perfused in retrograde fashion via the aorta with normal saline for 2 minutes. Post-residual blood drainage from the coronary arteries and tightening of the ligature, hearts were frozen after infusion of 5 ml of green fluorescent microspheres (Duke Scientific Corp., Palo Alto, CA, USA, suspended in saline), which was used to demarcate the risk zone from the normally perfused tissue. Twenty-four hours later, 3-mm thick sections obtained from the in 1% triphenyl tetrazolium chloride (TTC) in isotonic phosphate buffer, pH 7.4. This was followed by incubation in 10% formaldehyde to allow clear demarcation of the infarcted areas. Slices were examined under UV light (wavelength 366 nm) to delineate the borders between normal area and the risk zone. Finally, the normal area, risk zone, and infarcted area were traced onto an acetate sheet placer on a top glass plate, followed by scanning using Abode Photoshop 6.0 and measurement using Scion Image program. Infarct- and risk area volumes, calculated from the corresponding areas, were compounded followed by estimation of the percent of infarct to risk area ratio (%I/R) [11].

### Immunoblot analysis

Immunoblot analysis was performed as described previously using cardiac tissue homogenates [10]. Antibodies (Cell Signaling) used were rat Akt (1:1,000), P-Akt [1:500 (Ser473)], P42 MAPK/ERK (1:1,500), and P-P42MAPK/ERK [1:1,500 (Thr202/Tyr204)].

### Statistical analysis

Statistical analysis was performed using the SPSS 16.0 statistical software package (SPSS Inc., IL, and USA). Kolmogorov–Smirnov test was used for assessment of normal distribution among continuous variables. Group-wise comparison was done using *t*-test or one-way analysis of variance (ANOVA), whereas two-way ANOVA for repeated measurements was used to assess the effect of treatment status. All data, unless otherwise mentioned, were expressed as mean ± standard error of the mean (SEM). A *P* value of less than 0.05 was considered to be statistically significant.

## Abbreviations

IPC: Ischemic Postconditioning
I/R: ischemia/reperfusion
eNOS: endothelial nitric oxide synthase
TTC: triphenyl tetrazolium chloride
ANOVA: analysis of variance
SEM: standard error of the mean
IS: infarction sizes
AAR: area at risk

## References

[1] Ferdinandy P, Schulz R, Baxter GF (2007). Interaction of cardiovascular risk factors with myocardial ischemia/reperfusion injury, preconditioning, and postconditioning. Pharmacol Rev.

[2] Zhao ZQ, Corvera JS, Halkos ME, Kerendi F, Wang NP, Guyton RA, Vinten-Johansen J (2003). Inhibition of myocardial injury by ischemic postconditioning during reperfusion: comparison with ischemic preconditioning. Am J Physiol Heart Circ Physiol.

[3] Iliodromitis EK, Zoga A, Vrettou A, Andreadou I, Paraskevaidis IA, Kaklamanis L, Kremastinos DT (2006). The effectiveness of postconditioning and preconditioning on infarct size in hypercholesterolemic and normal anesthetized rabbits. Atherosclerosis.

[4] Wu N, Zhang X, Jia P, Jia D (2015). Hypercholesterolemia abrogates the protective effect of ischemic postconditioning by induction of apoptosis and impairment of activation of reperfusion injury salvage kinase pathway. Biochem Biophys Res Commun.

[5] Kupai K, Csonka C, Fekete V, Odendaal L, van Rooyen J, de W Marais, Csont T, Ferdinandy P (2009). Cholesterol diet-induced hyperlipidemia impairs the cardioprotective effect of postconditioning: role of peroxynitrite. Am J Physiol Heart Circ Physiol.

[6] Heart Protection Study Collaborative Group (2002). Mrc/bhf heart protection study of cholesterol lowering with simvastatin in 20,536 high-risk individuals: a randomised placebo-controlled trial. Lancet.

[7] Iliodromitis EK, Andreadou I, Prokovas E, Zoga A, Farmakis D, Fotopoulou T, Ioannidis K, Paraskevaidis IA, Kremastinos DT (2010). Simvastatin in contrast to postconditioning reduces infarct size in hyperlipidemic rabbits: possible role of oxidative/nitrosative stress attenuation. Basic Res Cardiol.

[8] Fan Y, Yang S, Zhang X, Cao Y, Huang Y (2012). Comparison of cardioprotective efficacy resulting from a combination of atorvastatin and ischaemic post-conditioning in diabetic and non-diabetic rats. Clin Exp Pharmacol Physiol.

[9] Fan Y, Yang S, Cao Y, Huang Y (2013). Effects of acute and chronic atorvastatin on cardioprotection of ischemic postconditioning in isolated rat hearts. Cardiovasc Ther.

[10] Patti G, Pasceri V, Colonna G, Miglionico M, Fischetti D, Sardella G, Montinaro A, Di Sciascio G (2007). Atorvastatin pretreatment improves outcomes in patients with acute coronary syndromes undergoing early percutaneous coronary intervention: results of the ARMYDA-ACS randomized trial. J Am Coll Cardiol.

[11] Iliodromitis EK, Gaitanaki C, Lazou A, Aggeli IK, Gizas V, Bofilis E, Zoga A, Beis I, Kremastinos DT (2006). Differential activation of mitogen-activated protein kinases in ischemic and nitroglycerin-induced preconditioning. Basic Res Cardiol.

[12] Li X, Zhao H, Wu Y, Zhang S, Zhao X, Zhang Y, Wang J, Wang J, Liu H (2014). Up-regulation of hypoxia-inducible factor-1alpha enhanced the cardioprotective effects of ischemic postconditioning in hyperlipidemic rats. Acta Biochim Biophys Sin (Shanghai).

[13] Kocsis GF, Pipis J, Fekete V, Kovács-Simon A, Odendaal L, Molnár E, Giricz Z, Janáky T, van Rooyen J, Csont T, Ferdinandy P (2008). Lovastatin interferes with the infarct size-limiting effect of ischemic preconditioning and postconditioning in rat hearts. Am J Physiol Heart Circ Physiol.

[14] Darling CE, Jiang R, Maynard M, Whittaker P, Vinten-Johansen J, Przyklenk K (2005). Postconditioning via stuttering reperfusion limits myocardial infarct size in rabbit hearts: role of erk1/2. Am J Physiol Heart Circ Physiol.

[15] Nishino Y, Webb IG, Davidson SM, Ahmed AI, Clark JE, Jacquet S, Shah AM, Miura T, Yellon DM, Avkiran M, Marber MS (2008). Glycogen synthase kinase-3 inactivation is not required for ischemic preconditioning or postconditioning in the mouse. Circ Res.

[16] Bell RM, Yellon DM (2003). Atorvastatin, administered at the onset of reperfusion, and independent of lipid lowering, protects the myocardium by up-regulating a pro-survival pathway. J Am Coll Cardiol.

[17] Hausenloy DJ, Tsang A, Yellon DM (2005). The reperfusion injury salvage kinase pathway: a common target for both ischemic preconditioning and postconditioning. Trends Cardiovasc Med.

[18] Schwartz LM, Lagranha CJ (2006). Ischemic postconditioning during reperfusion activates Akt and ERK without protecting against lethal myocardial ischemia-reperfusion injury in pigs. Am J Physiol Heart Circ Physiol.

[19] Obal D, Preckel B, Scharbatke H, Müllenheim J, Höterkes F, Thämer V, Schlack W (2001). One MAC of sevoflurane provides protection against reperfusion injury in the rat heart *in vivo*. Br J Anaesth.

[20] Cavallini DC, Bedani R, Bomdespacho LQ, Vendramini RC, Rossi EA (2009). Effects of probiotic bacteria, isoflavones and simvastatin on lipid profile and atherosclerosis in cholesterol-fed rabbits: a randomized double-blind study. Lipids Health Dis.

[21] Wolfrum S, Grimm M, Heidbreder M, Dendorfer A, Katus HA, Liao JK, Richardt G (2003). Acute reduction of myocardial infarct size by a hydroxymethyl glutaryl coenzyme a reductase inhibitor is mediated by endothelial nitric oxide synthase. J Cardiovasc Pharmacol.

